# Treatment strategies for hyperkalemia secondary to urethral obstruction in 50 male cats: 2002–2017

**DOI:** 10.1177/1098612X221127234

**Published:** 2022-11-09

**Authors:** Jessica M Jones, Jamie M Burkitt-Creedon, Steven E Epstein

**Affiliations:** 1 Veterinary Medical Teaching Hospital, School of Veterinary Medicine, University of California, Davis, Davis, CA, USA; 2 Department of Surgical and Radiological Sciences, School of Veterinary Medicine, University of California, Davis, Davis, CA, USA

**Keywords:** Feline idiopathic cystitis, insulin, dextrose-insulin ratio, electrolyte imbalance, emergency medicine, critical care medicine, sodium bicarbonate

## Abstract

**Objectives:**

The aims of this study were: to describe the potassium-lowering treatment strategies used to manage moderate-to-severe hyperkalemia in male cats with urethral obstruction (UO); to determine how much dextrose was required per unit of insulin to prevent hypoglycemia; to determine whether early initiation of a dextrose continuous rate infusion (CRI) prevented hypoglycemia; and to determine whether in-hospital mortality was associated with presenting plasma potassium concentration ([K^+^]).

**Methods:**

The medical records of male cats presenting with a [K^+^] ⩾7.0 mEq/l due to UO that had another [K^+^] measured within 6 h were reviewed retrospectively. All [K^+^] values within the first 6 h, blood glucose concentrations, treatments for hyperkalemia and survival to discharge were recorded. Analyses were performed to test for associations between dextrose:insulin ratios or method of dextrose administration and the development of hypoglycemia; and for presenting [K^+^] and mortality. Normally distributed groups of continuous data were compared with a *t*-test and categorical data were compared with a Fisher’s exact test.

**Results:**

Fifty cats were included. Mean presenting [K^+^] was 8.9 ± 1.0 mEq/l, while the mean final [K^+^] within 6 h was 6.6 ± 1.4 mEq/l. Forty-two (84%) cats were treated with intravenous fluids and 40 (80%) were treated with dextrose and insulin. Median dextrose:insulin ratio was 2 g/u (range 0.4–100). No dextrose:insulin ratio was found to protect against hypoglycemia, and 3/8 cats that became hypoglycemic had received ⩾2 g dextrose per unit of insulin. There was no association between the early initiation of a dextrose-containing CRI and avoidance of hypoglycemia. No association was found between presenting [K^+^] and mortality.

**Conclusions and relevance:**

While no specific dextrose:insulin ratio was found to protect against hypoglycemia, there is evidence that the commonly recommended dextrose:insulin ratio of 2 g/u may be inadequate in preventing hypoglycemia in every cat. Severity of hyperkalemia was not associated with mortality.

## Introduction

Hyperkalemia is a life-threatening electrolyte disturbance commonly seen in cats with urethral obstruction (UO). Hyperkalemia can cause life-threatening cardiac arrhythmias and generalized muscle weakness. A 2019 retrospective analysis of all-cause dyskalemia found that dogs and cats presenting with moderate-to-severe hyperkalemia had a significantly increased risk of death.^1^ Of the cats included in that study, 38% had UO.

A wide variety of methods is used to treat hyperkalemia secondary to UO in an emergency veterinary setting. Authors recommend different treatments, often in combination, including intravenous isotonic crystalloid fluid therapy (IVF); medications, including insulin with dextrose, sodium bicarbonate and adrenergic receptor agonists; and, ultimately, relief of the UO.^2,3^ A PubMed search on 3 July 2022 for ‘feline hyperkalemia veterinary’ revealed no primary literature addressing treatment strategies for hyperkalemia or their potential adverse effects in cats with naturally occurring UO. Potential concerns include the fact that, although insulin administration can cause hypoglycemia and hypoglycemic seizures, the risks associated with administering insulin to this population of non-diabetic cats has not been described.

The primary objective of this retrospective study was to describe the potassium-lowering treatment strategies used by clinicians managing male cats presenting to a university teaching hospital emergency service with moderate-to-severe hyperkalemia secondary to UO. Secondary objectives were to determine how much dex-trose was required per unit of insulin to prevent development of hypoglycemia; to determine whether early initiation of a dextrose continuous rate infusion (CRI) decreased the incidence of hypoglycemia; and to determine whether in-hospital mortality of any cause was associated with the presenting plasma potassium concentration.

## Materials and methods

The computerized medical record database of the Veterinary Medical Teaching Hospital at the University of California, Davis, was searched for all cats with a plasma potassium concentration ([K^+^]) ⩾7.0 mEq/l (ABL 705 and ABL 800; Radiometer Medical) between 1 January 2002 and 31 December 2017. The results of this search were manually reviewed. Cats were then included in the study if they had the following: ⩾2 [K^+^] measurements on the same point-of-care (PoC) analyzer within the first 6 h, the first of which revealed [K^+^] ⩾7.0 mEq/l; were male; at least 6 months old; and had hyperkalemia associated with UO diagnosed that day by physical examination findings of a firm, inexpressible urinary bladder with appropriate historical findings such as stranguria, pollakiuria, periuria or apparent dysuria.

Cats were excluded: if they had a past or current diagnosis of diabetes mellitus, insulinoma, hyperadrenocorticism or hypoadrenocorticism; if they were known to have received glucocorticoids within the prior 2 weeks or if they received them within the 6 h study window, since glucocorticoids can iatrogenically increase blood glucose by causing insulin resistance; if their UO was determined to be caused by neoplasia; or if their hyperkalemia and UO were not clearly related. Cats were not excluded for being referred to our hospital, as long as they fitted the inclusion criteria above.

Data collected from the medical records were documented in a standardized spreadsheet (Microsoft Excel). Data collected included: signalment; body weight; body condition score (BCS); cause of UO; presenting [K^+^] and blood glucose concentration (BG); all subsequent [K^+^] measured within 6 h of the first; all BGs recorded in the first 12 h; pertinent treatments administered during the first 6 h, including IV fluid therapy, sodium bicarbonate, insulin, dextrose, sympathomimetic drugs and calcium; method of obstruction relief; doses of insulin and dextrose administered within the first hour; additional doses of insulin and dextrose administered after the first hour; method of dextrose administration (intermittent bolus vs CRI vs both); timing of the initiation of dextrose CRI if one was used; and survival to hospital discharge. Time zero was defined as the time of the first [K^+^] measurement; the hospital’s PoC analyzer automatically time-stamped all results.

For ease of evaluation, all insulin and dextrose administered within the first hour were considered a single dose. If additional doses of insulin were administered beyond the first hour, only the BGs collected before the administration of the second dose of insulin were reported and analyzed. If a cat presented with hypoglycemia or did not have BG recorded following insulin administration, it was excluded from analysis regarding the development of hypoglycemia. The most likely cause of UO was determined based on the non-standardized diagnostic tests completed by the supervising clinician, and was categorized as feline idiopathic cystitis, bacterial cystitis, urolithiasis, neoplasia, foreign body or other. If diagnostic tests were not performed to determine the likely cause of UO, the cause was categorized as unknown. Feline idiopathic cystitis was diagnosed when comprehensive testing ruled out other causes. Bacterial cystitis was defined as a positive bacterial urine culture when ⩾10^3^ colony-forming units (CFU)/ml when urine was collected via cystocentesis, ⩾10^4^ CFU/ml when urine was collected via catheterization and ⩾10^5^ CFU/ml when urine was collected via free catch, as is standard practice for our laboratory.^4^

Urolithiasis was defined as organized mineral material identified within the bladder or urethra using either radiography or ultrasonography. Neoplasia was defined as a growth of the bladder or urethra causing UO identified ultrasonographically or by direct visualization at surgery or necropsy. A foreign body was defined as an organic or inorganic substance originating from outside the body causing a UO confirmed by direct visualization at time of removal or necropsy.

Normoglycemia was defined per our in-house, feline-specific reference intervals (RIs) of the PoC analyzer as BG of 80–127 mg/dl; a BG of <80 mg/dl was thus considered as hypoglycemia. A normal [K^+^] was defined per our in-house, feline-specific RI of the PoC analyzer as [K^+^] of 3.1–4.7 mEq/l. A normal rectal temperature was defined as 100.0–102.5°F (37.7–39.2°C). A heart rate (HR) <180 beats/min (bpm) was considered as bradycardia for this population of acutely ill cats.

### Statistical analysis

**Figure 1:**
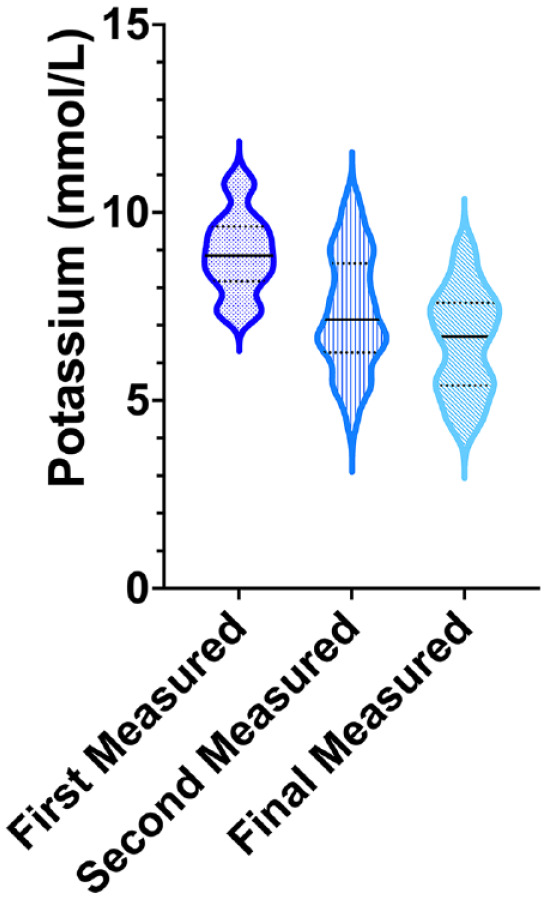
A violin plot representing the [K^+^] of 50 male cats with urethral obstruction at presentation compared with their second [K^+^] and their final [K^+^] within 6 h after collection of the initial [K^+^]. The width of the plot represents the number of cats that shared that [K^+^]. The solid horizontal line is the median [K^+^] at that sample. The dashed horizontal lines above and below the solid horizontal line represent the 75th and 25th percentiles, respectively

Descriptive statistics were used to report findings in this retrospective study. Normality of data was determined by a Shapiro–Wilk test. Normally distributed data are reported as mean ± SD, except in Figure 1, where normally distributed data are reported as median and interquartile range (IQR). Non-normally distributed data are reported as median (range). Groups of continuous data that were normally distributed were compared with a *t*-test and categorical data were compared with a Fisher’s exact test. A *P* value of <0.05 was considered to be statistically significant.

## Results

In total, 203 records of male cats with a [K^+^] of ⩾7.0 mEq/l were reviewed. Of these, 90 were not included because the cat did not have a second [K+] measured within 6 h. Of the remaining 113 records, 62 could not be included because the cats were not diagnosed with UO. The remaining 51 cats met the inclusion criteria. One cat was then excluded because its hyperkalemia could not be directly related to its UO. Therefore, 50 cats were included in the study. Forty-one cats presented to our emergency department for first-opinion care while nine were referred from other clinics with a diagnosis of UO.

### Signalment

The mean age at presentation was 5.5 ± 3.2 years (n = 47). Three cats were categorized in our system only as ‘adult’ in age. Forty-nine (98%) cats were castrated males and one was intact. Thirty-five cats (70%) were domestic shorthairs, six (12%) were domestic mediumhair, three (6%) were domestic longhair and two (4%) were Siamese. There were one of each (2%) of the following breeds: Maine Coon, Ragdoll, Manx and Persian. Median body weight was 6.3 kg (range 3.8–11.2).

### Physical examination findings

Mean BCS was 6.7 ± 1.3 (out of 9; n = 46). Mentation at presentation recorded in 49 cats was obtunded in 31 (63%), quiet in nine (18%), alert in five (10%) and stuporous or moribund in four (8%).

Hydration status was reported in 21 cats. Nine cats (43%) were estimated to be 5% dehydrated; nine (43%) were estimated to be 7–8% dehydrated; 2 (10%) were euhydrated; and one (5%) was estimated to be 10% dehydrated.

The penis was described in 17 cats, and every description (100%) was abnormal. Subjective description of the penile tissue included discoloration (erythematous, purple, black), presence of a urethral plug at the tip of the penis or, in one case (6%), redundant penile tissue obscuring the urethral orifice.

Rectal temperature was recorded in 43 cats. Thirty cats (70%) were hypothermic. Median rectal temperature was 37.4°C (range <32.2–39.8; 99.4°F [range <90–103.7]). HR was recorded for all cats. The median HR was 180 bpm (range 100–300). Nearly half (n = 24 [48%]) were bradycardic. Respiratory rate was recorded in 46 cats. Median respiratory rate was 36 breaths/min (range 15–120).

### Cause of UO

The cause of the UO could be determined from the medical record in 28/50 cats (56%). UO was likely due to feline idiopathic cystitis in 16 cats, urolithiasis in 10 and associated with urinary tract infection in two. No cat was diagnosed with foreign body or neoplasia as the cause of UO. Cause of the UO could not be determined based on medical record review in 22/50 cats (44%).

### Plasma potassium concentration

The mean value of the first recorded [K^+^] was 8.9 ± 1.0 mEq/l (n = 50). The mean value of the second [K^+^] collected in the 6 h study window was 7.3 ± 1.5 mEq/l (n = 50), while the mean final [K^+^] measured in the 6 h study period 6.6 ± 1.4 mEq/l (n = 50). Changes in [K^+^] over the 6 h study period are shown in Figure 1 and Table 1.

**Table 1:** Change in [K^+^] over time from first measured [K^+^] in 50 male cats with urethral obstruction that presented with a [K^+^] of ⩾7.0 mEq/l (time 0) following treatment at 1, 2, 4 and 6 h

Time of plasma potassium measurement (h)	No of cats	Plasma potassium concentration (mEq/l) vs time 0 (mean ± SD)
1	34	−1.6 ± 1.0
2	19	−2.3 ± 1.4
4	23	−2.7 ± 1.5
6	13	−2.6 ± 1.6

Some cats had [K^+^] measured more than twice in the 6 h study period and thus appear in this table at more than one time point

### Electrocardiographic findings

Forty-one cats underwent electrocardiography (ECG) monitoring at presentation to the emergency room. Cardiac rhythms included normal sinus rhythm in 10 (24%), absent P waves in nine (22%), tented T waves in seven (17%), sinoventricular rhythm in five (12%) or a combination of the above abnormal findings in 10 (24%).

### Description of medical treatments to address hyperkalemia

A variety of medical treatments were used to reduce [K^+^]. These included IVF, dextrose with insulin, dextrose without insulin and sodium bicarbonate. Sympathomimetic drugs were not used to decrease [K^+^] in any cat. No cat received insulin therapy without receiving concurrent dextrose supplementation.

IVF therapy was used in 42 (84%) cats. It was the sole medical treatment in four (8%) cats. Dextrose with insulin was used in 40 (80%) cats. It was the sole medical therapy in two (4%) cats. Dextrose without insulin was used in three (6%) cats. Twenty-two cats that were administered dextrose received at least some portion of the dextrose as a CRI following the initial bolus. Sodium bicarbonate was used in 22 (44%) cats. Sodium bicarbonate was not administered as the sole medical therapy in any cat. Many cats were treated with a combination of the above therapies. A list of the medical treatments administered is shown in Table 2. One cat’s hyperkalemia was not treated with medical therapy, but only by relief of the obstruction on presentation to the emergency room.

**Table 2:** Medical treatment strategies for 50 cats with hyperkalemia secondary to urethral obstruction

Medical treatment for the reduction of hyperkalemia	No of cats (%)
IVF, dextrose, insulin	21 (42)
IVF, dextrose, insulin, sodium bicarbonate	14 (28)
IVF, dextrose, sodium bicarbonate	2 (4)
IVF, sodium bicarbonate	1 (2)
IV fluids only	4 (8)
Dextrose, insulin, sodium bicarbonate	4 (8)
Dextrose and insulin only	2 (4)
Dextrose, sodium bicarbonate	1 (2)
None	1 (2)

IVF = intravenous isotonic crystalloid fluid therapy

Although not used as a method to lower the [K^+^], IV calcium was administered in 36 (72%) cats; 35 (97%) of these cats were treated with calcium gluconate and one (3%) was treated with calcium chloride.

### Dextrose:insulin doses and development of hypoglycemia

Presenting BG was recorded in 48 cats. The median presenting BG was 191.5 mg/dl (range 33–522). Hypoglycemia on presentation was noted in four (8%) cats, none of which had received any therapy prior to presentation. Hyperglycemia on presentation was noted in 33 (69%) cats.

In the 40 cats treated with insulin and dextrose, the median insulin dose administered in the first hour was 0.17 unit/kg (range 0.07–0.5). The median dextrose dose administered in the first hour was 0.45 g/kg (range 0.1–1). In one cat that received insulin and dextrose, the dose of dextrose was not recorded; thus, this cat was not included for consideration of the dextrose–insulin relationships that follow. The median dextrose:insulin ratio administered in the other 39 cats was 2 g dextrose to 1 unit insulin (range 0.4–100 g/unit). Of the cats that received insulin and dextrose, three presented hypoglycemic and were subsequently excluded from analysis regarding the development of hypoglycemia. Of the remaining 36 cats that received insulin and dextrose, five were lacking BG data (two had no BG measured for the visit and three had only a presentation BG but none recorded following insulin administration) and were therefore excluded from analysis regarding development of hypoglycemia.

Fifteen of the 31 (48%) remaining cats that presented non-hypoglycemic developed hypoglycemia within 12 h of receiving an initial dose of dextrose and insulin for the treatment of hyperkalemia. Of these 15 cats, seven developed hypoglycemia only after receiving an additional dose of insulin between hours 2 and 6, and thus their BGs were not analyzed further. The BGs of the remaining eight cats that became hypoglycemic were only considered for analysis until they received their second dose of insulin. The median BG of these eight cats was 63 mg/dl (range 33–75).

Thirty-one cats were evaluated for the time to development of hypoglycemia following insulin and dextrose administration, and eight (26%) developed hypoglycemia within 12 h. Of these eight cats that developed hypoglycemia after only a single dose of insulin, six (19%) became hypoglycemic within 6 h, with a median time to hypoglycemia of 5 h (range 2–6). Two additional cats that received insulin only within the first hour of treatment became hypoglycemic between hours 6 and 12. The median time to hypoglycemia for all eight cats that developed hypoglycemia after receiving insulin during only the first hour of treatment was 5.5 h (range 2–11).

Of the 31 cats receiving insulin and dextrose that were evaluated for the development of hypoglycemia, nine (29%) received dextrose supplementation only as a bolus with the first dose of insulin. Of these cats, three (33%) became hypoglycemic in the first 12 h; one each at 3.5, 5 and 11 h. The dextrose:insulin ratios administered to these cats were 2.5, 1 and 2.1 g dextrose per unit of insulin, respectively. The range of dextrose:insulin ratios administered to cats that did not become hypoglycemic was 2–100 g dextrose per unit of insulin.

Of the three cats that presented hypoglycemic and received insulin and dextrose boluses, two were placed on an isotonic crystalloid supplemented with 5% dextrose. The other did not receive a dextrose-containing CRI. Of the two cats that were placed on CRIs containing dextrose, one did not receive any further insulin or dextrose boluses. Its potassium steadily improved and no further episodes of hypoglycemia were recorded. The other received further doses of insulin and dextrose at unknown times and doses. This cat experienced a second hypoglycemic event 3 h after the collection of its first [K^+^]. The cat that did not receive a dextrose-containing CRI did not receive any further doses of insulin or dextrose. Its potassium steadily improved and it did not have any further recorded hypoglycemic events.

### Development of hypoglycemia in cats receiving a dextrose CRI

Of the six cats that developed hypoglycemia within 6 h, four (67%) had received a dextrose CRI from the time of insulin administration; of the two cats that developed hypoglycemia between 6 and 12 h, one received a dextrose CRI starting at the time insulin was given. Unfortunately, it was impossible to determine the total doses of dextrose administered to most cats by medical record review. However, whether a dextrose CRI was initiated within the first hour of treatment was not associated with development of hypoglycemia (*P* = 0.66).

### Return of normokalemia

Only 6/50 cats achieved normokalemia in the 6 h study window. Presenting [K^+^] for these six cats ranged from 7.2 to 9.9 mEq/l, and the final [K^+^] ranged from 4.1 to 4.7 mEq/l. Five of the six cats received IVF therapy; 5/6 received sodium bicarbonate; and 3/6 received insulin and dextrose. Two cats received dextrose, one as a bolus and one as a CRI, without insulin. Of the three cats that received insulin and dextrose, none received a second dose of insulin outside of the first hour.

### Relief of UO

Relief of urinary tract obstruction was achieved in all 50 cats. Forty-seven cats (94%) underwent retrograde urethral catheterization. In three cats, retrograde urethral catheterization was unsuccessful. Thus, 2/50 (4%) cats had their obstruction temporarily relieved via decompressive cystocentesis prior to definitive surgical intervention and one (2%) had its obstruction relieved via tube cystostomy.

### Mortality

Forty of 50 (80%) cats survived to discharge. Of the 20% that did not survive to discharge, 2/10 (20%) experienced cardiopulmonary arrest. Cardiopulmonary resuscitation (CPR) was performed in one cat and return of spontaneous circulation was achieved. This cat was then euthanized prior to discharge. A diagnosis of sepsis was made on necropsy. CPR was not attempted in the other cat that experienced cardiopulmonary arrest. A clinical diagnosis of pulmonary edema was made in this cat based on frothy, blood-tinged fluid issuing from the mouth. One cat (1/10 [10%]) suffered respiratory arrest and was euthanized shortly after. The remaining seven non-survivors (70%) were euthanized due to severity of disease and/or client financial concerns. The mean presenting [K^+^] of cats that survived to discharge was not different than that of cats that did not (8.9 ± 1.1 mEq/l vs 8.9 ± 0.8 mEq/l; *P* = 0.96).

## Discussion

The primary objective of this study was to describe the methods used to treat moderate-to-severe hyperkalemia in a population of cats with UO. Most cats received IVF therapy as part of their initial treatment; this treatment lowers [K^+^] by increasing glomerular filtration rate, thus enhancing potassium elimination, and by dilution. The next most frequent treatment used in our population was insulin and dextrose in combination. These treatment strategies are among those commonly recommended for treatment of hyperkalemia in cats with UO.^2,3^

Administration of exogenous insulin stimulates the sodium–potassium ATPase pumps on cell surfaces, promoting the net movement of extracellular potassium into the cells.^5^ The administration of dextrose without exogenous insulin is thought to stimulate the release of endogenous insulin from the beta islet cells of the pancreas, causing the same upregulation of the sodium–potassium ATPase pumps.^5^

Nearly half the cats were treated with sodium bicarbonate as part of the potassium-lowering strategy. Several theoretical mechanisms have been used to justify the use of sodium bicarbonate in hyperkalemic patients: that alkalization increases the activity of the hydrogen–potassium exchangers on cell surfaces, directly moving potassium ions into the cell, and increases the activity of the sodium–hydrogen ion exchanger on skeletal muscle cells, thus driving the sodium–potassium ATPase pump to move potassium into the cell in exchange for sodium.^6,7^ Additionally, any metabolic alkalosis created by administration of sodium bicarbonate upregulates distal tubular potassium channels and increases renal potassium excretion.^7^ Despite these proposed physiologic mechanisms, evidence for use of sodium bicarbonate to treat hyperkalemia is lacking. A recent Cochrane meta-analysis revealed limited evidence to support the use of sodium bicarbonate therapy to treat acute hyperkalemia in people.^8^ It has been suggested that sodium bicarbonate may be useful in life-threatening hyperkalemia when metabolic acidosis is present.^6^There are several disadvantages of sodium bicarbonate that make its routine use controversial. The handling of sodium bicarbonate in the body leads to an equimolar production of carbon dioxide (CO_2_). In patients with poor minute ventilation, administration of sodium bicarbonate can exacerbate acidemia. Even in patients with adequate minute ventilation, an increase in CO_2_ can occur and diffuse into the cells causing a paradoxical intracellular acidosis. Additionally, sodium bicarbonate is a hypertonic solution that can lead to an undesired increase in intravascular volume, hypernatremia and hyperosmolality. A decrease in ionized calcium concentration may be seen following administration of sodium bicarbonate, as an increase in pH causes calcium to bind albumin.^9^ Given that sodium bicarbonate was not used alone in any cat in this study, it is unclear whether it was appropriate or effective. It remains unclear whether sodium bicarbonate should be used to treat severe hyperkalemia in cats with UO. Considering many cats experiencing UO may have moderate-to-severe metabolic acidosis that could itself benefit from buffered fluid therapy, it may be interesting to explore the use of isotonic bicarbonate solution as a fluid therapy choice for these cats.

No cats in this study were treated with sympathomimetic drugs, even though authors recommend them for treatment of hyperkalemia in small animals.^2,3^ It is possible that clinicians elected not to use sympathomimetics because of cardiovascular instability or the potential for subclinical cardiomyopathy, considering sympathomimetics too risky. It is equally likely that an institutional bias exists against the use of sympathomimetics for treatment of hyperkalemia in our hospital. Sympathomimetics lower [K^+^] by stimulation of the sodium–potassium ATPase pumps, causing an intracellular shift in potassium.^6^ Of note, sympathomimetic therapy in conjunction with insulin and dextrose is first-line therapy for all-cause hyperkalemia in people.^8^ Frequency of sympathomimetic use to treat hyperkalemia in cats in the general veterinary emergency community is unknown.

One secondary objective of this study was to determine how much dextrose was required per unit of insulin to prevent development of hypoglycemia in this population of non-diabetic cats. Given the low numbers (n = 8) of cats that could be evaluated for the development of hypoglycemia, meaningful statistical analyses could not be performed regarding a dextrose:insulin ratio that protected against evolution of hypoglycemia. Unfortunately, the inconsistent timing and frequency of BG measurements, in combination with the variability in timing and reporting of dextrose therapies, made it impossible to determine useful relationships among timing, method, and dose of dextrose and insulin over the 6 h study period.

Interestingly, of the nine cats in which a definitive dextrose:insulin dose could be determined, three became hypoglycemic; two of these cats had received >2 g dextrose for each unit of insulin (dextrose:insulin ratios of 2.1 and 2.5 g/u). This finding suggests that the previously recommended ratio of 2 g dextrose for every 1 unit of insulin administered may not be sufficient to avoid hypoglycemia in all cats.^10,11^ To our knowledge the primary source of the recommendation for dextrose:insulin ratio of 2 g dextrose to 1 unit insulin is an abstract that appears not to have made it to full peer-reviewed publication.^12^ In the most current human literature, there is evidence that a higher dextrose to insulin ratio of 3 –5 g/u should be used to treat all-cause acute hyperkalemia.^13^

Some veterinary authors have recommended administering 0.5 U/kg insulin and 2 g dextrose/unit insulin administered to treat hyperkalemia.^14,15^ However, no primary reference appears to support this dose. Given the variability of recommendations for dextrose dosing for this indication, and the fact our population contained cats that developed hypoglycemia despite receiving ⩾2 g dextrose/unit insulin, it seems reasonable to recommend a minimum of 3 g dextrose per unit insulin (0.3 g/kg when administering a dose of 0.1 u/kg regular insulin) in non-diabetic cats for treatment of hyperkalemia. Further study is needed to determine the optimal strategy, as this study is small and may not represent the hyperkalemic UO population as a whole.

Another secondary objective of this study was to determine whether use of a dextrose CRI, as opposed to only a single injection of dextrose, decreased the incidence of hypoglycemia. Of the cats treated with insulin and dextrose, there was no significant difference in the development of hypoglycemia between cats that received dextrose bolus therapy immediately followed by a dextrose CRI and those that received bolused dextrose therapy only. Causes for this finding are likely multifactorial. It is possible that the dose of dextrose some cats received as a bolus was insufficient to prevent hypoglycemia from occurring, even though a dextrose CRI was started immediately. In the abstract by Loeb et al,^12^ the authors compared the difference in decrease of serum potassium and development of hypoglycemia in four dogs administered either 2 g dextrose for every 1 unit of insulin or 4 g of dextrose for every 1 unit of insulin. These doses of dextrose were divided, given half as an initial bolus and half as a CRI over the following 2 h. The authors of that study noted that while the lower ratio (2 g dextrose for every 1 unit of insulin) resulted in a more substantial decrease in [K^+^], it also led to greater occurrences of hypoglycemia than the higher dextrose:insulin ratio. The cause for the discrepancy in [K^+^] drop is unknown. Further prospective research is required to investigate the usefulness and optimal dosing of dextrose CRI in the treatment of hyperkalemic cats receiving insulin.

The final secondary objective of this study was to determine whether there was an association between initial [K^+^] and survival to hospital discharge. There was no difference in the presenting [K^+^] between survivors and non-survivors, all of which presented with [K^+^] of ⩾7.0 mEq/l. This finding may be related to selection bias: to be included, cats were required to have two [K^+^] measured within the initial 6 h. Many cats with an initial plasma [K^+^] ⩾7.0 mEq/l may have died or been euthanized before a second plasma [K^+^] could be measured, which would skew our population to a less sick group of cats that had owners with more financial means and thus the ability to provide more medical support. Additionally, it may be that the relationship between [K^+^] and likelihood of survival is non-linear. It is possible that the decrease in plasma potassium concentration over time in response to treatment is a more effective predictor of survival than the initial concentration.

Current reported survival-to-discharge frequency for cats with UO is 89–95%.^16–18^ In contrast, the current study had a survival frequency of 80%. This difference is likely because all cats in this study presented with a [K^+^] of ⩾7.0 mEq/l and were thus likely sicker than the general population of cats with UO. The degree of hyperkalemia in this population likely reflected chronicity of obstruction. Cats that remain obstructed long enough to develop moderate-to-severe hyperkalemia may also be more likely to experience other negative consequences such as acute kidney injury or post-obstructive diuresis. Any of these complications may prolong hospitalization and impact an owner’s ability to continue care financially.

The underlying cause of UO could be determined in only 56% (n = 28/50) of cats due to lack of diagnostic work-up. Of those cats only 57% (n = 16/28) were assigned a diagnosis of FIC. This finding contrasts with more recent studies, which describe FIC to be the cause of UO in 71–90% of cats.^19–21^ This disparity is likely because the medical records in 44% (n = 22/50) of cats did not contain adequate information to determine a definitive diagnosis. Many of these cats likely had obstructive FIC.

This study had several limitations, most related to its retrospective nature. Specific treatment timings, doses and combination of treatments were not standardized, but were instead administered at the discretion of the attending clinician; many medical records lacked specific doses and rates of dextrose, for example, or may have contained typographical errors, as is possible with the cat that was recorded to have received 100 g of dextrose/unit of insulin. Additionally, the reassessment of the cats [K^+^] and any further interventions were not standardized. Treatments such as IVF therapy and sodium bicarbonate may have been administered to cats at presentation for reasons other than hyperkalemia; the retrospective nature of our study made it impossible to determine the treating clinicians’ motivations. Furthermore, we were unable to assess cats for pre-existing kidney dysfunction that could have altered their potassium clearance. Moreover, the small number of cats included in this study may have created a type 2 error in our results and conclusions. The option for euthanasia in the veterinary setting confounds the findings, as clinicians may have discussed euthanasia more frequently in cats with a higher presenting [K^+^] due to the expectation of a protracted clinical course and cost of treatment. It is possible that many cats with UO were euthanized prior to collection of a second [K^+^], which biases the population of cats reported here. Euthanasia also confounds the ability to accurately assess for an association between mortality and [K^+^].

## Conclusions

The most common medical treatments used to reduce [K^+^] over the study period were IVF therapy and insulin with dextrose. No dextrose:insulin ratio used in this population predictably prevented the development of hypoglycemia; however, this study showed that a dextrose:insulin ratio of 2 g/u is inadequate in preventing hypoglycemia in some cats, regardless of whether a dextrose-containing CRI is administered. Additionally, this study did not find an association between presenting [K^+^] and mortality. Avenues for further prospective research include analysis of dextrose:insulin ratios that prevent the development of hypoglycemia, the utility of isotonic bicarbonate solution as IVF therapy in acidemic cats with UO, and whether the presence of moderate-to-severe hyperkalemia contributes to mortality in cats with UO.
